# Genetic Fuzzy Based Scalable System of Distributed Robots for a Collaborative Task

**DOI:** 10.3389/frobt.2020.601243

**Published:** 2020-12-23

**Authors:** Anoop Sathyan, Kelly Cohen, Ou Ma

**Affiliations:** Department of Aerospace Engineering, University of Cincinnati, Cincinnati, OH, United States

**Keywords:** collaborative control, genetic fuzzy system, decentralized control, intelligent systems, machine learning, cable robot

## Abstract

This paper introduces a new genetic fuzzy based paradigm for developing scalable set of decentralized homogenous robots for a collaborative task. In this work, the number of robots in the team can be changed without any additional training. The dynamic problem considered in this work involves multiple stationary robots that are assigned with the goal of bringing a common effector, which is physically connected to each of these robots through cables, to any arbitrary target position within the workspace of the robots. The robots do not communicate with each other. This means that each robot has no explicit knowledge of the actions of the other robots in the team. At any instant, the robots only have information related to the common effector and the target. Genetic Fuzzy System (GFS) framework is used to train controllers for the robots to achieve the common goal. The same GFS model is shared among all robots. This way, we take advantage of the homogeneity of the robots to reduce the training parameters. This also provides the capability to scale to any team size without any additional training. This paper shows the effectiveness of this methodology by testing the system on an extensive set of cases involving teams with different number of robots. Although the robots are stationary, the GFS framework presented in this paper does not put any restriction on the placement of the robots. This paper describes the scalable GFS framework and its applicability across a wide set of cases involving a variety of team sizes and robot locations. We also show results in the case of moving targets.

## 1. Introduction

We introduce a new scalable framework of Genetic Fuzzy System (GFS) for training a distributed system of robots to work collaboratively to achieve a common goal. The system needs to be trained such inter-robot communication about their actions are not required. This reduces the communication overhead of the system. Also, the team of robots should be able to achieve the goal regardless of the number of robots in the team. Thus, the trained robots are expected to collaborate regardless of the size of the team. This is extremely important in fault scenarios where a robot can breakdown and become inactive in the system, as well as in scenarios where more robots are added to provide more strength and efficiency. This work builds on our previous research (Sathyan and Ma, 2019) that was limited to teams consisting only upto 5 robots that were located only in a symmetric topology.

Collaborative robotics technology is being applied in numerous applications, such as moving objects (Tuci et al., 2018), observing moving targets (Khan et al., 2016), helping human workers in collaborative tasks (Realmuto et al., 2017; Roveda et al., 2019), coordinated search activities (Baranzadeh and Savkin, 2017; Chamanbaz et al., 2017), etc. Unlike many existing multi-robot control strategies, our work described in this paper involves a decentralized team of robots. This means that the robots are all independently controlled without relying on any centralized controller. The major advantage with a decentralized system is that it is more fault tolerant. Any fault in a centralized controller affects the entire system, whereas in a decentralized system, faults in individual robots are localized and does not necessarily affect the entire system (Zhang and Mehrjerdi, 2013). Moreover, such faults can easily be rectified by removing or replacing the faulty robot in the team.

Recently, decentralized strategies are being explored in robotics. Such strategies have been used for training each leg of a hexapod robot to achieve stability when standing (Sartoretti et al., 2019a). Each agent in the system controls one leg of the hexapod system. Another work involves a robot team consisting of two robots where the robots are able to understand each others intentions without explicit communication (Losey et al., 2020). This is achieved by defining a role for each of the robots. In our system, in addition to the robots being decentralized, they are not required to communicate with each other, reducing the communication overhead. Our framework is also scalable in that the number of robots in the system can be increased or decreased without having to retrain the robots.

Most of the robots used in industrial applications today are pre-programmed for the specific task and do not provide the flexibility to modify the system to work for other tasks (Villani et al., 2018). Machine learning architectures can be used as decision making models for robots to provide more flexibility while also enabling other benefits including robustness to uncertainties, adaptability to different applications and improved efficiency. They also provide the ability to fuse information coming in from different sources to make better decisions for performing tasks in an informed, efficient manner. Learning decentralized controllers for large network of mobile robots with sparse communication links have been presented in a recent work (Tolstaya et al., 2020). Training decentralized controllers that can be used on different team sizes for scalability have been presented for a construction problem. This work presented a neural network based methodology for a construction related task where the robots were trained on a team of four robots and later tested on larger teams up to 20 robots (Sartoretti et al., 2019b).

Genetic Fuzzy System (GFS) is a machine learning framework that can be used to model intelligent robot controllers. In a GFS, the parameters of the Fuzzy Logic System (FLS) are optimized for the specific task using Genetic Algorithm (GA). Such GFSs have proven to be extremely successful in various applications including task assignment and planning (Sathyan et al., 2016), simulated air-to-air combat (Ernest et al., 2016), athlete movement prediction (Sathyan et al., 2019b), etc. GFS framework was also used for our previous work on a problem where the robots were placed along a regular polygon with the same objective of bringing the common effector to any arbitrarily defined position within the polygon (Sathyan and Ma, 2018, 2019; Sathyan et al., 2018, 2019a).

The linguistic rulebase of GFSs make it more interpretable compared to other machine learning techniques. GFSs are tuned using GA and hence the cost function does not necessarily have to be differentiable. Through the operations of crossover and mutation, the individuals in GA are modified over several generations to search for the optimal set of GFS parameters—membership functions and rules—that minimize the defined cost function. This makes it a form of reinforcement learning as the robots are trained to take the optimal control actions to maximize a reward or in this case, minimize the cost function. The cost function acts as reinforcements for training the robot agents. It is important to note that the cost function in our GFS framework is different from the reward function defined for Q-learning (Watkins and Dayan, 1992). The cost function in GA takes into account the overall performance of the system during a training episode, whereas the rewards in Q-learning are usually calculated for each individual action.

FLSs provide designers the ability to model the relationship between the inputs and outputs using linguistic IF-THEN rules. When this relationship is known apriori, such expert knowledge can be incorporated using the rulebase of the FLS. This approach has been used in several robotics applications. This will usually involve minimal tuning of the FLS parameters using trial and error. FLSs have been used for controlling mobile robots that have to navigate within dynamically changing cluttered workspace (Omrane et al., 2016), path planning for navigating nano-robots through blood vessels for drug delivery (Mobadersany et al., 2015), assisting human operators in heavy industrial tasks (Roveda et al., 2019).

Even though expert knowledge can be used to design FLSs, it is highly useful to have an efficient process for tuning FLS paramters automatically. The manual design becomes increasingly difficult as the number of inputs and outputs increase. The ability to automatically tune the FLS parameters is also useful for applications where the relationship between the input and output variables is not well-known. The ANFIS (Adaptive Network based Fuzzy Inference System) (Jang, 1993) framework, that uses a combination of the backpropagation algorithm and recursive least squares, has been a popular approach for training Sugeno-FLSs. However, ANFIS is a supervised learning methodology and would require a dataset of state-action pairs for training. On the other hand, GA can be used for training FLSs without any state-action ground truth data. The GFS can be trained based on the cost function designed for the application.

In this paper, we extend on our previous work (Sathyan and Ma, 2019) to design a scalable GFS framework for a reinforcement learning problem involving a team of stationary, homogenous robots. Mamdani FLS is considered in this work. The problem involves training a team of robots that are connected to a common effector through elastic cables. The objective is to bring the common effector to any desired position within the workspace of the multi-robot system. In our previous works, the robots were positioned along the vertices of regular polygons and the results were shown only for 3- and 5-robot cases. It was noticed that training for the 5-robot system is much more complex compared to the 3-robot system. In this paper, we introduce a scalable GFS framework that allows the system to scale to any number of robots without any additional training. We also extend the capability to general scenarios where the robots are placed at arbitrary positions.

In this work, each GFS is trained to model the control actions of a robot in the team at each instant, such that the series of collaborative actions taken by the robot in the team helps with bringing the common effector to the desired target position quickly. The same GFS model is shared across all robots in the team. This makes the training process very efficient and also provides much needed scalability to the system, as the same GFS can be used when adding more agents to the team without additional training.

## 2. Problem Statement

**Figure 1:**
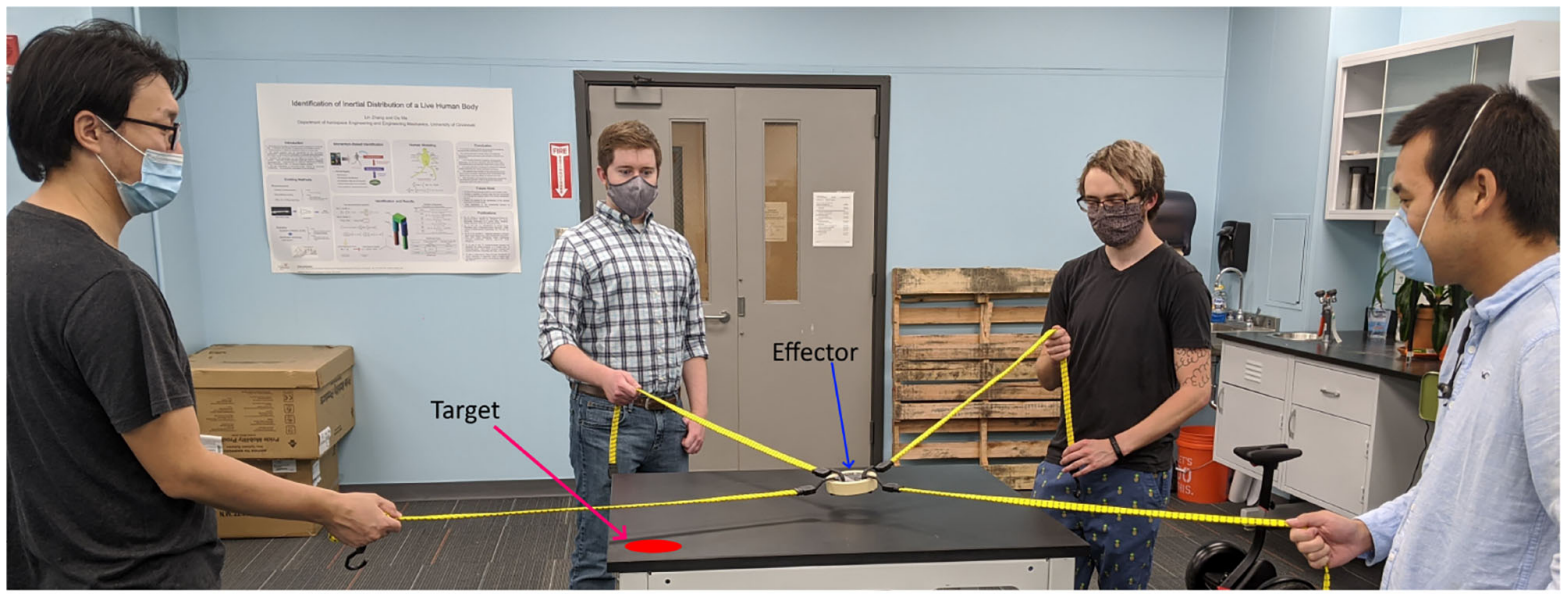
A team game that requires the participants to work together for success. The participants are tasked with the objective of bringing the common effector to the target position by controlling the elastic cables connected to the effector.

A sample dynamics problem is considered in this work to test the applicability of the proposed GFS framework. It takes its inspiration from a fun collaborative activity that involves people pulling on cables to control the position of the commonly connected effector in order to bring it toward a target position, as shown in Figure 1. The participants do not communicate with each other. Each participant pulls or releases the cable to control the common effector. They have to work collaboratively to bring the effector to the target position. This activity highlights the ability of human teams to learn and devise strategies that allows for the successful and efficient completion of the collaborative task. So, it is an appropriate choice of a challenging task to test the proposed GFS framework to train a decentralized system of robots to work collaboratively toward the common goal of bringing the effector to the target. Although this collaborative activity may be rather easy for humans, it would be quite challenging for robots.

The objective of the multi-robot team is to devise a strategy to work collaboratively while remaining decentralized to bring the effector to any target position. Each robot controls its own cable. An added constraint is that the robots do not communicate with each other. This means that each robot do not use any information on the states of its partner robots to make its decisions. During the training process, the performance of the system as a whole will be considered to evolve the team of robots. Thus, even though there is no communication between the robots, there will be some implicit understanding between the robots that results from the general strategy they use to achieve the goal.

Each robot is assumed to have a DC motor that is attached to a spool for winding its cable. The cables are pulled or released by controlling the voltage of the DC motor. The GFS controller of each robot controls the voltage of its DC motor which in turn controls the cable. Together as a group, each robot action affects the position of the common effector. It is understood that the workspace of the robots is the convex hull connecting the robot positions. This makes sense as the effector cannot be at equilibrium at any point outside of this polygon.

## 3. Fuzzy Logic Systems (FLS)

Unlike Boolean logic, fuzzy logic deals with degrees of truth rather than a binary True or False. This can be generalized to degrees of membership to different sets or membership values. This gives a fuzzy boundary between different sets rather than a crisp division. This process of converting a crisp value to fuzzy membership values that represent the membership level of an input value to each of the fuzzy sets is called fuzzification. Fuzzification happens based on how the membership sets are defined for each of the input and output variables. Another important aspect of an FLS is the rulebase which is a set of linguistic rules that gives an intuitive relationship between the inputs and outputs. An example for an FLS rule used for calculating tip is *If Food is Good and Service is Good then Tip is Good*.

**Figure 2:**
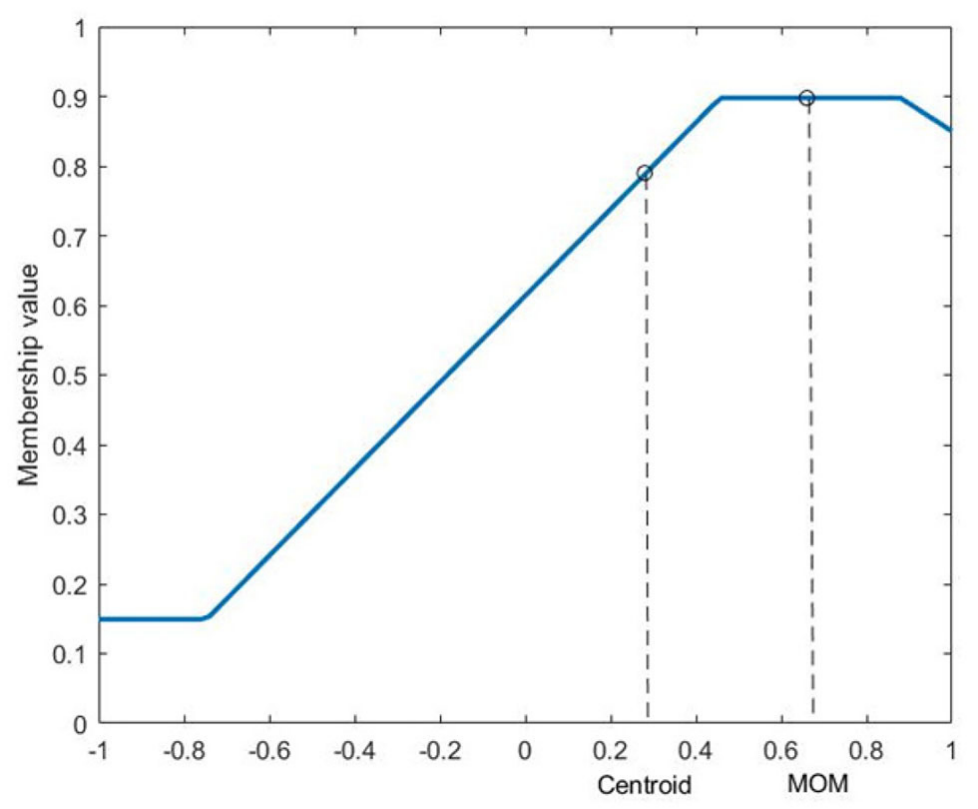
Centroid and MOM defuzzification outputs. The x-axis is the output variable.

In order to convert the output of an FLS back to a crisp value, each rule in the rulebase of the FIS needs to be evaluated and then the resultant aggregate solution should be defuzzified. The aggregate solution after rule evaluation will be a region defined in membership space of the output variable, similar to the one shown in Figure 2. There are different defuzzification strategies used (Yager and Zadeh, 2012), the most popular of which is the centroid defuzzification. Centroid defuzzification gives the x-value of the centroid of the area which seldom reaches close to the extremities. This issue can be resolved by increasing the range of the output membership functions. Mean of maximum (MOM) defuzzification can also be used to obtain defuzzifed values that span the width of the output range. MOM defuzzification outputs the mean of the x-values that have the highest membership value, as shown in Figure 2.

For small-scale FLSs, the rulebase could possibly be defined using expert knowledge. But, this becomes difficult for larger FLSs. It is also difficult when the relation between the inputs and outputs is not known and needs to be learned. It is necessary to augment the FLS with learning capabilities for such applications. GA is a very effective tool for training FLSs for the specific application. These GFSs can learn from the training scenarios based on the cost function defined to ensure that all the design requirements are satisfied.

In the GFS framework, the set of parameters to be tuned include the boundaries of the membership functions and the set of rules in the rulebase. For some applications, GA is also used to tune the shape of the membership functions although in most cases, it is safe to assume triangular and trapezoidal membership functions. GFS framework can be used to tune different types of membership functions depending on how they are defined. Triangular membership functions are defined using the x-coordinates of the three vertices. Symmetric triangular membership functions can be defined using the center of the base of the triangle and its width. Gaussian membership functions are defined using the mean (center) and standard deviation. In this work, we consider only triangular membership functions.

## 4. Multi-Robot Problem

This section extends the dynamics analysis from our previous work (Sathyan and Ma, 2019) to asymmetric multi-robot topologies. For the sake of uniformity, we will follow the same notation system as in Sathyan and Ma (2019).

Let ***r***_*Ei*_ be the vector connecting the effector to robot *i*, and let *p*_*i*_ be the length of the cable wound around the spool of this robot. Thus, the total length of the cable attached to robot *i* will be *r*_*Ei*_ + *p*_*i*_. Let *l*_0_ be the equilibrium length of all the elastic cables and *k* be their spring constant.

**Figure 3:**
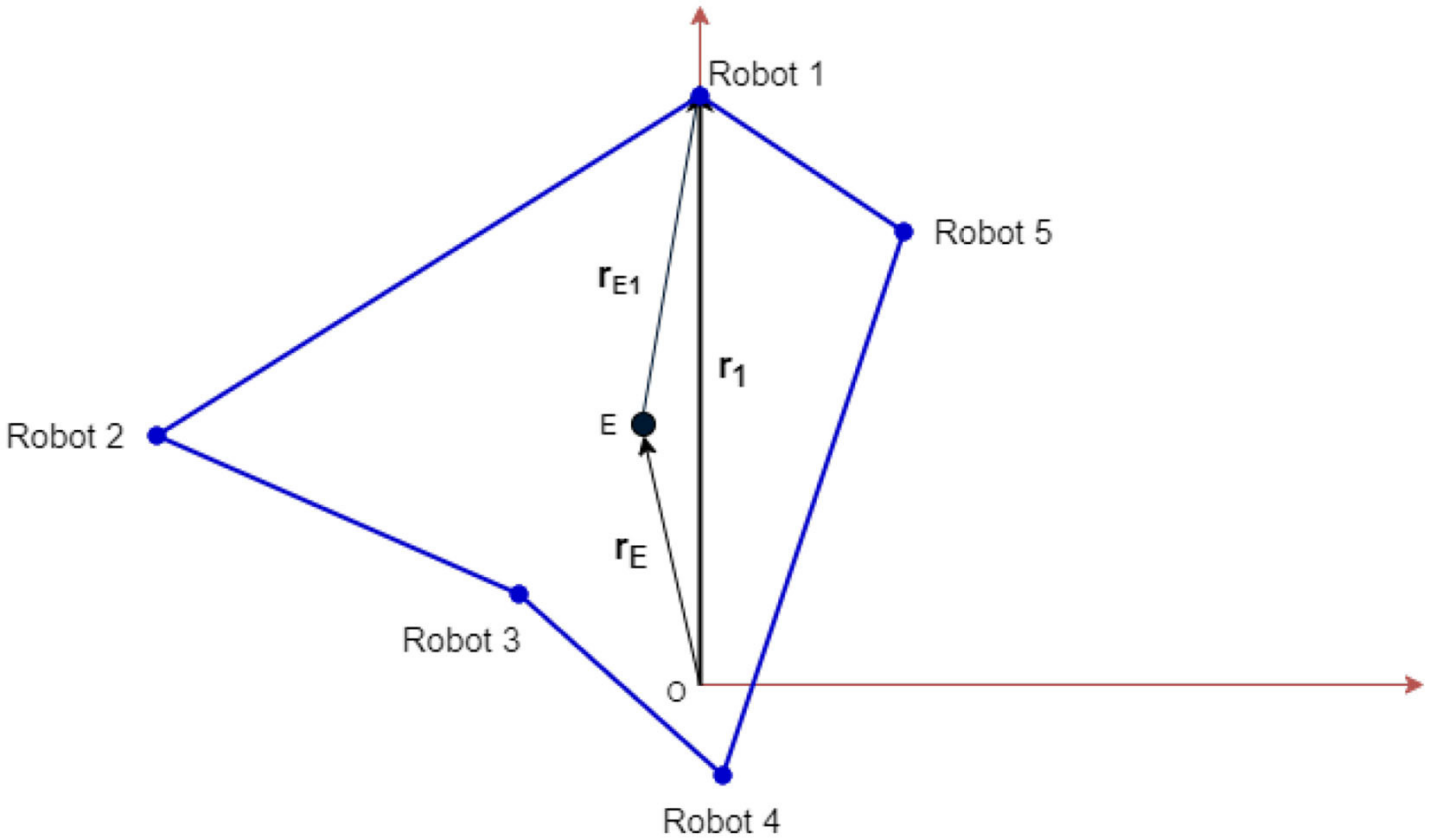
Schematic showing the vector notations. The 5 robots are placed arbitrarily. The common effector that is connected to each robot is denoted as E. This representation can be generalized for an *n*-robot system.

Figure 3 shows a visual representation of the different vectors involved for an asymmetric topology, where the robots are placed along the vertices of an irregular polygon. The tension on the elastic cable attached to robot-*i* will act along the unit vector, r^Ei, and is given by,

(1) Ti=k(rEi+pi-l0)r^Ei=k[(pi-l0)r^Ei+rEi]

***T***_*i*_ can be rewritten as,

(2) Ti=k[(pi-l0)r^Ei+ri-rE]

Assuming the effector has mass *m* leads to the following relation.

(3) $$\begin{array}{r} \overset{n}{\underset{i}{\sum }}T_{i}=m{\overset{¨}{r}}_{E} \end{array}$$

Since the robots are not located along a regular polygon, $\underset{i}{\sum }r_{i}\neq 0$. Substituting Equation (2) into Equation (3) gives the following governing equation for the effector under equilibrium condition. Here, *n* refers to the number of robots in the system.

(4) k∑in((pi-l0)r^Ei+ri)-nrE=mr¨Ewhere r^Ei=ri-rE|ri-rE|

Equation (4) is a vector equation of motion of the common effector, E. The motion of the effector relates to the pull on the cables connecting the robots to the effector. As noted earlier, the objective of the robots is to control the effector through their cables to bring the effector to the target position. Thus, the objective of the system can be written as

(5) minimize J=∫0Tdist(t)dtsubject to k∑in((pi-l0)r^Ei)-nrE=mr¨E

Here, dist(t) refers to the distance between the common effector and the target at time *t*. Since we are assuming the robots to be fixed to a location and actuated using DC motors, each robot has only one joint driven by its DC motor. For the simulations, the specifications of an actual DC motor were used to define the range of the different motor variables, such as torque and angular velocity. The GFS controller of each robot models its DC motor will be trained to determine the optimal control voltage, *V*_*i*_(*t*) at each time step *t* for achieving the common goal of the robot team. The electro-mechanical equations corresponding to the DC motors are given below.

(6) $$\begin{array}{r} p_{i}=r_{s}\theta_{i} \end{array}$$

(7) $$\begin{array}{rc} K_{T}I_{i}-T_{i}r_{s}-b{\dot{\theta }}_{Gi} & =J{\ddot{\theta }}_{Gi} \end{array}$$

(8) $$\begin{array}{rc} L\frac{dI_{i}}{dt}+I_{i}R & =V_{i}-K_{e}{\dot{\theta }}_{Gi} \end{array}$$

(9) $$\begin{array}{rc} I & =\frac{K_{T}}{G_{r}}T_{Gi} \end{array}$$

Equations (6)–(9) provide the relation between the motor voltages, V, and the length of the cables, *p*. The motors inside the different robots are assumed to be the same and hence share the same specifications. The variables and parameters related to the motor include the gear ratio (*G*_*r*_), torque constant of the motor (*K*_*T*_), back-emf constant (*K*_*e*_), torque output to the spool (*T*_*G*_), the motor current (I), and voltage (V). The term $b{\dot{\theta }}_{G}$ refers to the damping torque. The variables related to the spool include its angular velocity (θ_*G*_), radius (*r*_*s*_) and moment of inertia (*J*). Equations (4)–(9) give the relationship between the voltages of the motors associated with the different robots, as determined by the corresponding GFS, and the motion of the common effector. Each robot action is thus affected by the voltage decided by its GFS, which in turn affects the motion of the effector. Thus, these robots can be trained to work as team such that their individual actions result in achieving the goal of bringing the effector to the target.

## 5. GFS Framework for Scalability

This section describes the methodology of applying GFS for developing systems of scalable robots. The system will be trained for a team of 5 robots and then tested on a generalized system of *n* robots without any additional training. The robots are assumed to be located at arbitrary positions and can interact with the effector only through the elastic cable connecting the common effector to each robot.

In this work, GFS controllers directly control the voltage of the joint motor of each robot which in turn pulls or releases the attached cable accordingly. Thus, the system of robots can work together to control the effector to bring it to the desired position on the table. Equations (4), (7), and (8) provide the governing equations of the dynamic system.

The GFS controller has four inputs: (1) the difference between the effector-robot distance and the target-robot distance, (2) the angle between the effector-robot vector and the target-robot vector, (3) velocity of the effector along the x-axis, and (4) velocity of the effector along the y-axis. In order to have the ability to be scalable in terms of the size of the robot team, all robots are modeled to have the same internal GFS controller. This means if the same inputs are provided to the robots, the robot actions will be the same. However, since the inputs to the GFS are relative to the robot frame of reference, the output actions suggested by the GFSs can be different for the same effector states.

The use of the same GFS model across all robots helps with adding more robots to a system without changing any of the internal robot controller parameters. To train the multi-robot team for this task of bringing the effector to any target position, the robots are first trained on scenarios involving 5 robots. Here, the number 5 is simply arbitrarily chosen.

The use of the same GFS controller for all homogenous robots provides several advantages to this system:

1. This provides scalability to the system. Since the internal control logic is the same, more robots can be added to the system or removed from the system without any retraining.
2. The number of parameters that need to be tuned is much less compared to a system of different controllers with same number of robots. The training for a 5-robot system was done within 2 h.
3. Since the inputs to the controllers are relative to the robot reference frame, the robots are able to make their own distinct decisions.

Although this approach has several such advantages, it is limited to homogenous system of robots. In heterogenous systems where individual robots have different capabilities, the GFSs provide an advantage in that the rulebase and membership functions can be tuned separately. In heterogenous systems, the robots will have different capabilities, however, the rules governing their actions can be the same. Thus, we propose to share the same rulebase across all individual robots. The membership functions will be different. This means, for heterogenous systems, only the membership functions need to be tuned as more robots are added to the team. We plan to explore this in our future work.

In order for the robots to achieve the common goal of bringing the effector to any arbitrarily defined target position, the system of robots need to be trained first on a set of chosen scenarios. Each scenario is defined using the locations of the robots and the position of the target. A set of validation scenarios can also be defined to check for overfitting. After each generation of GA, the GFS can be validated by using the best GFS on the validation scenarios in order to spot any overfitting. These validation scenarios are separate from the training scenarios and similar performance in both the training and validation set is a sign that the GFS is learning properly rather than fitting just to the training scenarios. Each scenario is run for a maximum of 20*s*.

The following function is defined as the cost function. This trains the team of robots to reduce the distance between the effector and the target quickly while also satisfying the physical constraints of the system.

(10) $$\begin{array}{r} C=\int_{0}^{T_{max}}\text{dist}\left(t\right)dt+P\left(T_{max}-t_{stop}\right) \end{array}$$

*T*_*max*_ refers to the maximum time, set as 20*s*, and *t*_*stop*_ refers to the time at which an episode of the simulation is forced to stop. The simulation can stop either when the time reaches maximum time, *T*_*max*_, or when atleast one of the physical constraints defined for the system is not satisfied. These physical constraints include (1) the length of the cable to be <2 m, (2) the angular velocity of the motor should be <195 rpm, and (3) the torque on the motor to be <1.6 Nm. The *P*(*T* − *t*_*stop*_) term is used to penalize cases where any of these constraints are violated. This ensures the trained system inherently understands the physical constraints of the system. *P* acts as a penalization factor. It can be a large positive value. In this work, we have set *P* = 50. *dist*(*t*) is the distance between the effector and the target at any instant *t*. Since the training is done on several scenarios, GA will try to the minimize the mean of the cost values across these training scenarios.

**Figure 4:**
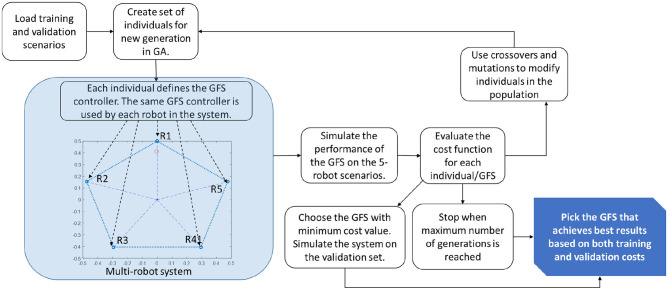
Schematic showing the training process in GFS. The GFS is trained on scenarios with 5 robots. The same GFS is used to control all robots, R1–R5.

The training process is shown in Figure 4. GA involves a population of individuals. At the beginning, the population is randomly generated. Each individual is a vector of tunable parameter values of the GFS. These include the membership function boundaries and the consequents of the rules in the rulebase of the GFS. The rulebase will include all combinations of the different membership functions. Each individual in GA relates to a GFS and since the same GFS is shared across all robots, each individual in the population refers to an entire team of robots. Thus, each individual can be used to simulate the dynamics and evaluate the cost function on the training scenarios.

The cost function would be representative of the ability of the system in achieving the goal. The lower the cost value, the better. The individuals with better cost values have higher likelihood of getting selected into the next generation. The individuals with high cost values have higher chance of getting eliminated from the population. This process of evolution continues for a predefined maximum number of generations. After each generation, the best GFS is also evaluated on the validation set to check for overfitting. Once GA converges, the best individual that performs well on both the training and validation cost is chosen to design the multi-robot system. This trained system is then tested on a series of test scenarios to check its generalization capability to ensure that the system works on a wide variety of scenarios. These new scenarios will includes changes to the size of the team, the location of the robots as well as the targets.

**Figure 5:**
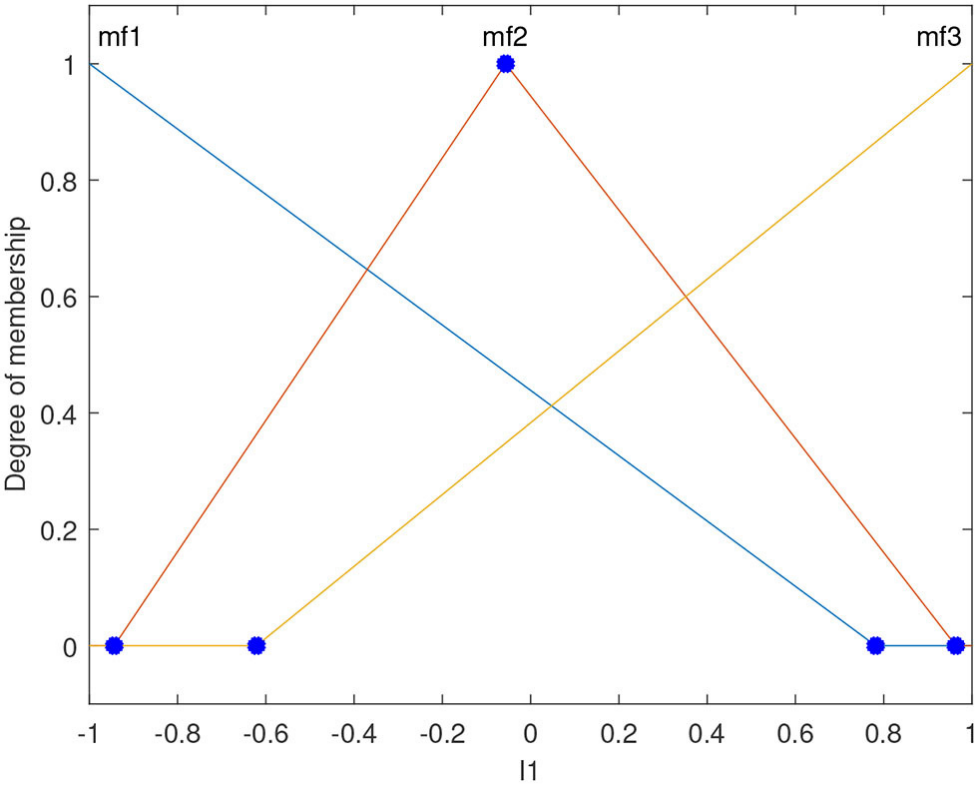
The format of the input membership functions of the GFS. The blue dots are tuned using GA. GA also tunes the membership function boundaries of the output as well as the rulebase of the GFS.

The input and output variables of the GFSs are modeled using triangular membership functions. For the input variables, GA is used to tune the three membership function boundaries for each input variable. These points are marked in blue as shown in Figure 5. The membership functions, mf1 and mf3, are assumed to peak at the two extremes of each variable. Five triangular membership functions are used to define the output variable, and the x-values of all the vertices of each triangle are tuned using GA. This means that GA tunes 15 parameters of the output membership functions for each robot.

Regarding rulebase of the GFS, since each of the four inputs are defined using three membership functions, there will be 3^4^ = 81 rules to represent all possible combinations of the membership functions across all inputs. Since the same GFS controller is copied across all robots, the training process involves tuning a total of 116 parameters. Each individual in GA will be a 116-element vector.

GA is setup in MATLAB with the options shown in Table 1. The crossover, mutation and selection functions are set to their default values in MATLAB. The GA with these options train the GFS to be capable of achieving the common goal for a wide range of scenarios, as described in the next section.

**Table 1:** GA options.

**Options**	**Value**
Number of generations	100
Population size	50
Stall generation limit	20
Number of variables	116
Crossover function	Scattered
Mutation function	Gaussian
Selection function	Stochastic uniform

## 6. Results and Discussion

The robots are trained to bring the effector to the target position. Each robot is controlled by its trained GFS module. MOM defuzzification is used by the GFSs.

The system was trained on 5-robot scenarios. The results presented in this section shows the effectiveness of the methodology by applying the trained GFS on various cases with different number of robots in the team. The results are both symmetric and asymmetric topologies.

### 6.1. Symmetric Topology

**Figure 6:**
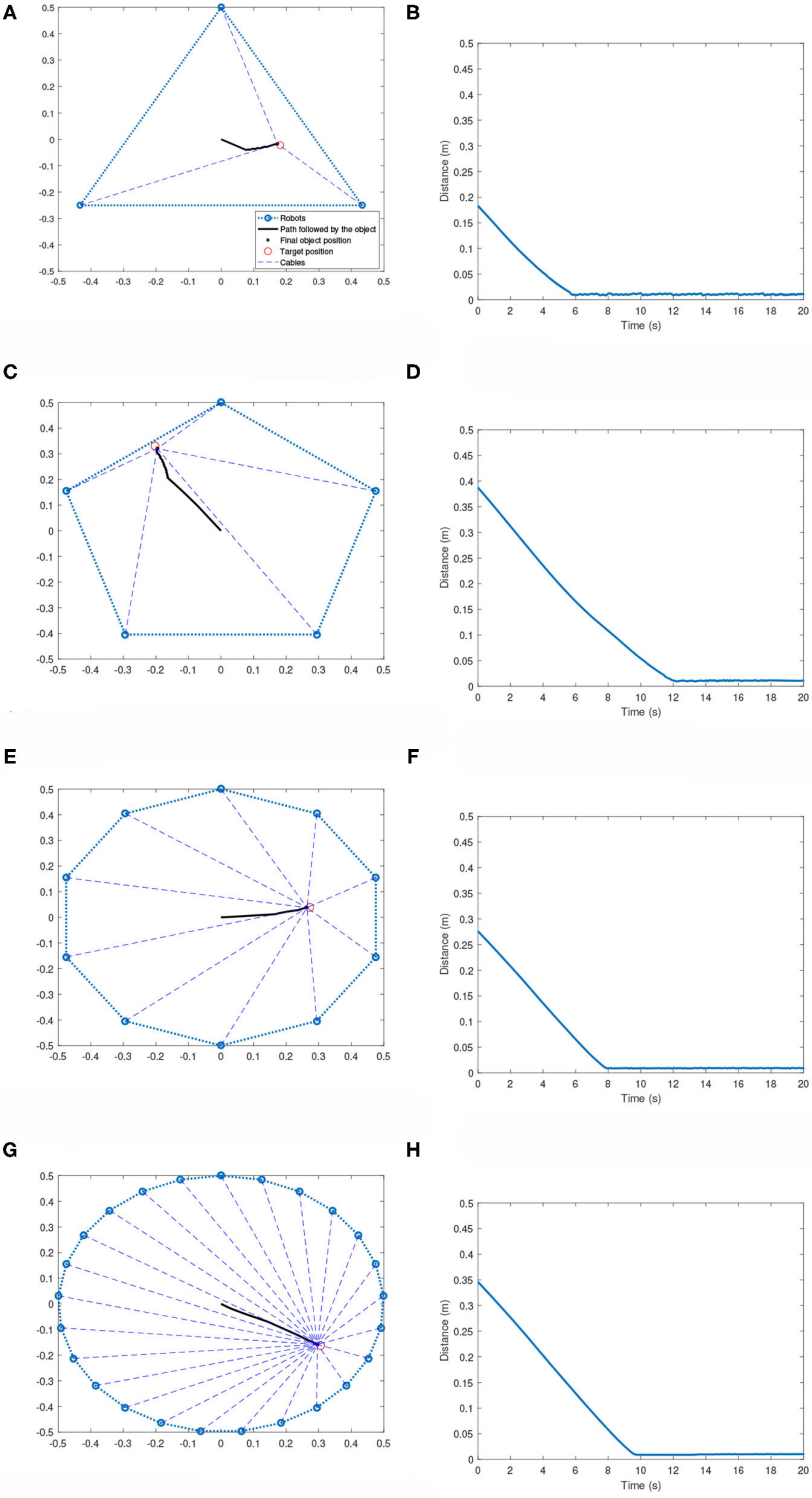
Multi-robot systems with robots following a symmetric topology. **(A)** 3-robot case: Effector's path toward the target. **(B)** 3-robot case: Distance plot. **(C)** 5-robot case: Effector's path toward the target. **(D)** 5-robot case: Distance plot. **(E)** 10-robot case: Effector's path toward the target. **(F)** 10-robot case: Distance plot. **(G)** 25-robot case: Effector's path toward the target. **(H)** 25-robot case: Distance plot.

In the symmetric topology, the robots are placed along the vertices of a regular polygon. The robots, after training on the 5-robot cases, are able to bring the effector to the corresponding target positions regardless of the size of the team. Figure 6 shows the performance of the system for 3, 5, 10, and 25-robot cases. As can be seen, the system is able to bring the effector very efficiently and quickly to the target region. The target region is defined as a circle of 2 cm radius around a target position. This is a very important result, as it shows that multi-robot systems for homogenous robots can be trained on cases with smaller sized teams, and then later be scaled to more complicated cases involving larger teams.

In our previous work (Sathyan and Ma, 2019), we had described some limitations, such as increased complexity of training as the number of robots are increased. Moreover, in the previous work, the GFSs had to be trained again when adding more robots. These limitations have been solved in this work by sharing the same GFS across all robots in the team. This also ensures that robots can be added or removed from the team without any additional training.

**Figure 7:**
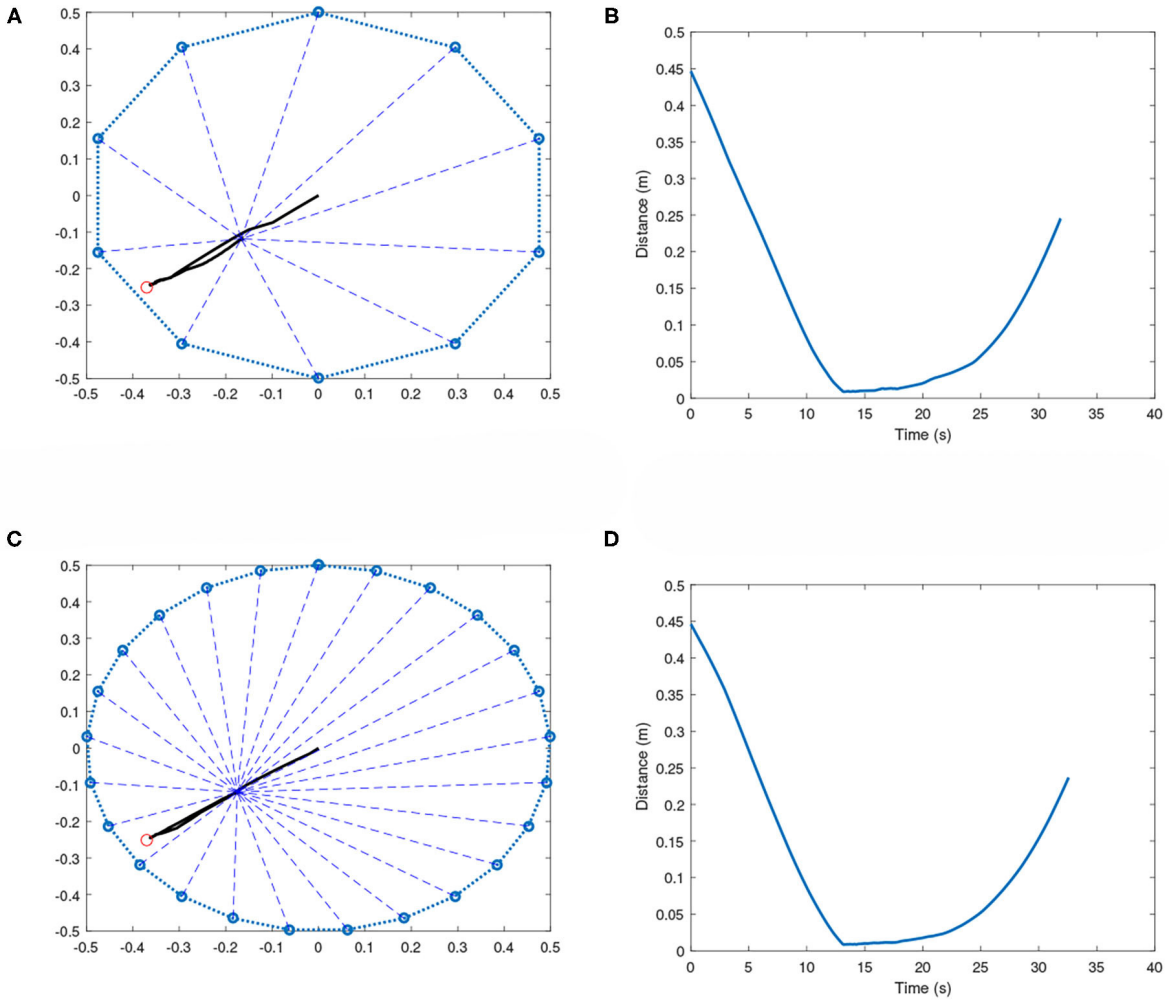
Symmetric multi-robot systems with target close to the boundary. **(A)** 10-robot case: Effector's path toward the target. **(B)** 10-robot case: Distance plot. **(C)** 25-robot case: Effector's path toward the target. **(D)** 25-robot case: Distance plot.

It is noticed when the target position is close to the boundary of the polygon, the team of robots is unable to keep the effector at the target region for long. In such cases, the effector is brought to the target and then tends to move away until a constraint has been broken. This phenomenon can be seen in Figure 7. This could be occurring due to the following reasons:

1. When the effector reaches the target position, which is very close to the boundaries, the tension forces *kx* along the different cables produces a lot of imbalance. This is because the number of tension vectors away from the boundary is much higher than the ones directed toward the boundary. This makes it very difficult to maintain equilibrium.
2. The GFSs directly control the voltages of the actuators in the respective robots, and not the angular velocities of the DC motor. This produces additional complexity in bringing the angular velocities of the spools to zero.
3. The elastic cable is modeled only as spring with no damping capability. The addition of damping parameter to the cables could reduce the chances of oscillations, which could help with keeping the effector in such complex positions (close to the boundary) for longer.
4. The training is performed for a maximum time *t* = 20*s* for each scenario. Increasing this maximum time during training may ensure that the effector stays at the target position for longer.

Table 2 summarizes the performance of systems with different team sizes. Each of the team sizes are run for 100 randomly defined scenarios and the figures of merit shown in the table are calculated. The scenarios were ran for a maximum time of 20 s. The mean and maximum refer, respectively to the mean and maximum over the 100 scenarios. The table shows the mean and max times to bring the effector to the target region (arrival time), mean and max cost values evaluated using Equation (10) and the mean and max of the Steady State Error (SSE). In the scenarios that were tested, we notice that in rare instances when the target is too close to the boundary of the control region, it becomes difficult to settle within the target region. This is why for some cases, the maximum SSE is >2 cm. However, even in these cases, the robots managed to bring the effector to the target region before drifting away. Based on the 100 test scenarios for each team size, it is noticed that the trained robots were successful in bringing the effector to the target region in all scenarios.

**Table 2:** Results for different team sizes with symmetric topology.

**Team size**	**Mean arrival time (s)**	**Mean cost**	**Mean SSE (m)**	**Max. arrival time (s)**	**Max. cost**	**Max. SSE (m)**
3	13.698	1.080	0.012	17.994	3.5782	0.074
5	9.716	1.458	0.009	16.925	3.208	0.021
10	9.584	1.803	0.011	16.110	3.504	0.031
25	9.436	1.742	0.011	16.097	3.521	0.044

It can be seen that the mean cost mostly increases as the number of robots are increased. This can be attributed to the fact the control region becomes larger as more robots are added. Hence, the target could be placed further from the starting position for larger team causing the cost value to increase.

Our previous works (Sathyan and Ma, 2018, 2019; Sathyan et al., 2018) were limited to a maximum team size of five, and it was also observed that the training got increasingly difficult with the size of the team. Successful completion of the task was achieved only on 90% of the scenarios tested. The proposed GFS framework, in this work, is shown scalable to any team size with computing power to simulate the dynamic system being the only limitation. The robots were able to achieve the goal for all the scenarios that were tested. This methodology works for different team sizes without any additional training. Of course, if heterogenous robots are added to the team some additional training will be needed. This methodology works even when the robots are located arbitrarily, as we will see next.

### 6.2. Asymmetric Topology

**Figure 8:**
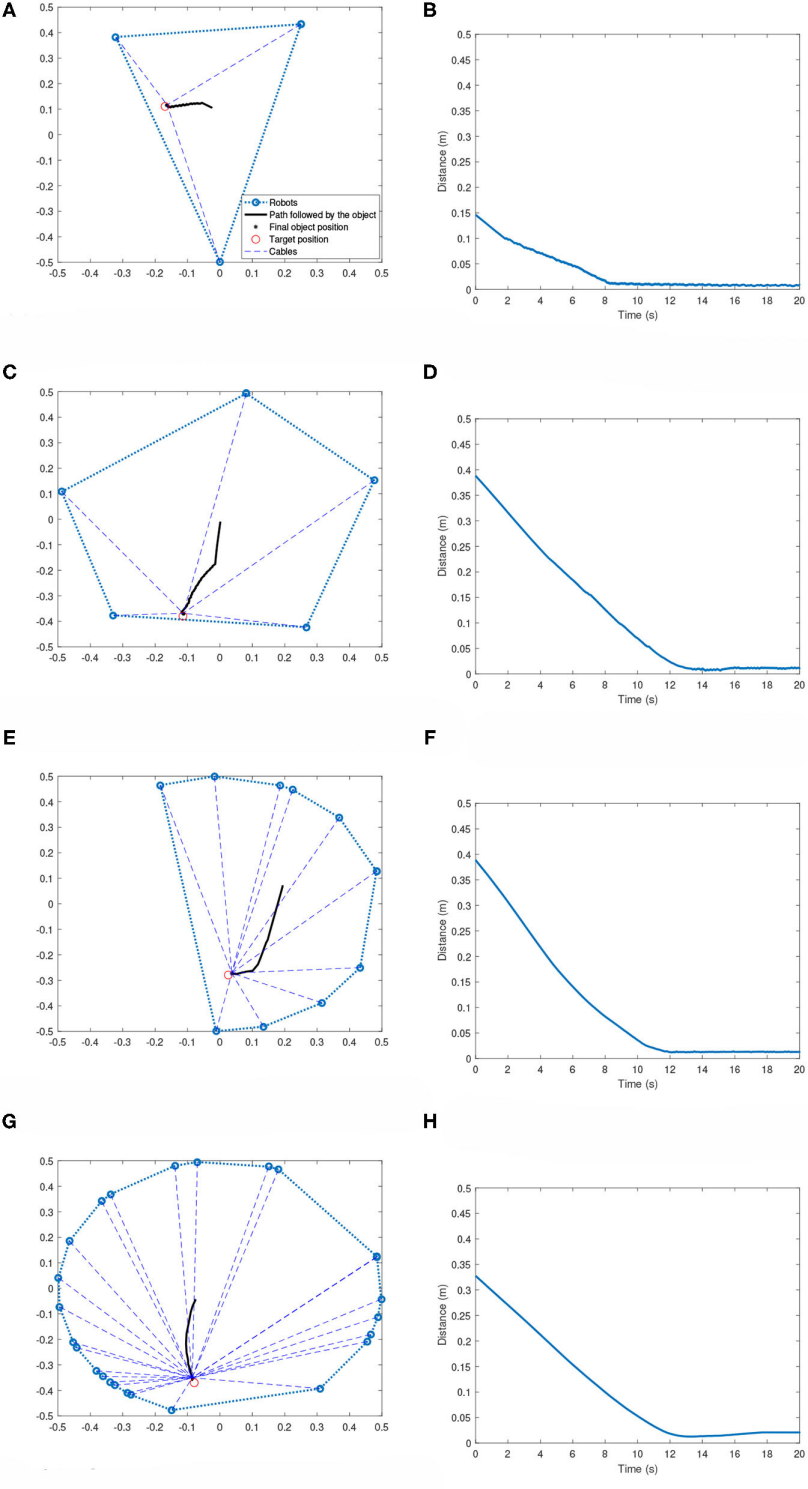
Multi-robot systems with robots located at arbitrary positions. **(A)** 3-robot case: Effector's path toward the target. **(B)** 3-robot case: Distance plot. **(C)** 5-robot case: Effector's path toward the target. **(D)** 5-robot case: Distance plot. **(E)** 10-robot case: Effector's path toward the target. **(F)** 10-robot case: Distance plot. **(G)** 25-robot case: Effector's path toward the target. **(H)** 25-robot case: Distance plot.

Figure 8 shows the performance of the system when robots assume an asymmetric topology. No additional training is done for these cases. The effector always starts from the equilibrium position, which will be centroid of the workspace in all cases. Just like the symmetric case, the robots are able to achieve the goal of bringing the effector to the target very efficiently.

Table 3 summarizes the results obtained after testing on 100 random scenarios for each of the team sizes. Each scenario is run till maximum time of 20 s. Both the robot positions and the target positions were changed for each scenario. It can be seen that compared to the results presented in Table 2, the settling times are slightly higher for the asymmetric cases. The robots were successful in bringing the effector to the target region in all the test cases showing the effectiveness of the methodology. Similar to the asymmetric topology, there are few instances, when the target is too close to the boundary, where the effector starts to drift outside of the target region.

**Table 3:** Results for different sized teams with asymmetric topology.

**Team size**	**Mean arrival time (s)**	**Mean cost**	**Mean SSE (m)**	**Max. arrival time (s)**	**Max. cost**	**Max. SSE (m)**
3	15.145	0.747	0.008	16.998	2.185	0.019
5	13.66	1.504	0.009	18.996	3.421	0.025
10	10.376	1.713	0.011	16.819	3.461	0.018
25	11.230	1.818	0.010	17.001	3.504	0.037

The fact that the system works well even when the robots are positioned arbitrarily means that the system is very robust to change in position of the robots. This means robots can be added or removed from the system without affecting its performance. Thus, the same GFS can be used even in scenarios where faulty robots need to be removed from the team. Of course, in such cases, the workspace of the modified system will be smaller.

### 6.3. Moving Target

**Figure 9:**
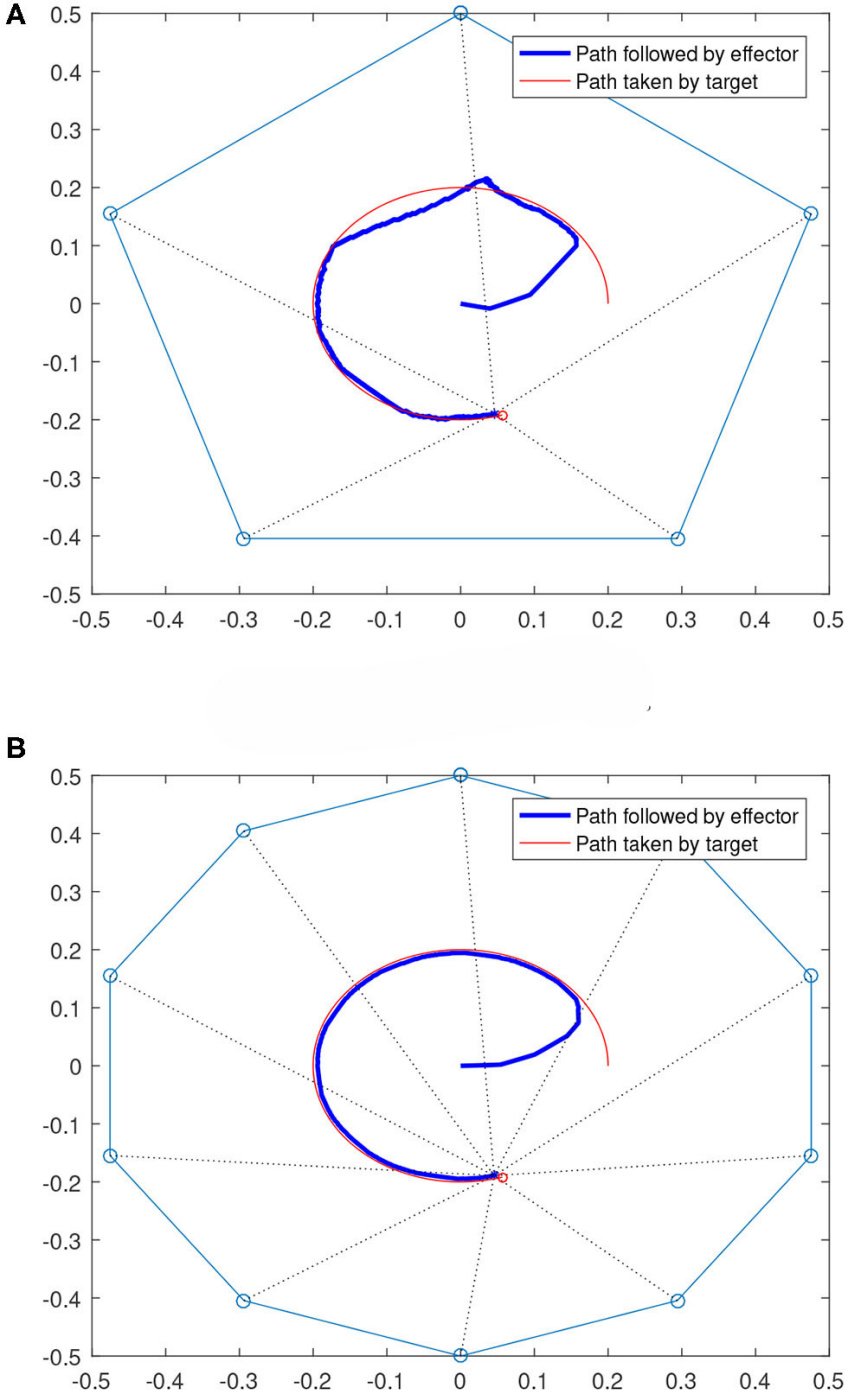
Multi-robot systems following moving targets. **(A)** Moving target case with 5 robots. **(B)** Moving target case with 10 robots.

We also considered cases where the targets are moving. Figure 9 shows two scenarios where the targets are moving along a circular path. For both 5 and 10 robot cases shown in Figure 9, the team is able to keep the effector to follow the target after starting from the origin (0,0). It can also be noticed that the path followed in the 10-robot cases is smoother and closer to the target's path than the 5-robot case. This is expected as the 10-robot system has more degrees of freedom and hence provides more flexibility in following a moving target.

## 7. Conclusions

In our earlier work (Sathyan and Ma, 2019), we applied the GFS framework to a decentralized system of collaborative robots for teams consisting up to 5 robots for symmetric topologies. In this paper, we presented a new scalable GFS framework for the same application that can be scaled to work regardless of the number of robots in the team. This works for asymmetric topologies as well. Due to the homogeneity of the robots, they can each be modeled using the same GFS controller. This ensures scalability. Since the inputs to each GFS are measured relative to each robot, the decisions made by each GFS can be different. The trained system was shown on various scenarios with teams ranging in size from 3 to 25 robots. The results for cases when the robots are located in asymmetric fashion were also presented.

Although the system was trained only on cases involving 5 robots distributed in a symmetric topology, the resulting controllers were shown to work even for asymmetric topologies with different team sizes. This shows that this GFS framework can be trained on simpler and smaller teams and then be scaled to more complex, larger sized teams. It also shows that the system does not have to be retrained when more robots are added to or removed from the team. Thus, the system can work, with a reduced workspace, even when multiple robots malfunction.

After extensive testing a range of testing scenarios, it is noticed that the trained system of robots is very efficient in bringing the effector to the target position. The lack of dependence on centralized controller and inter-robot communication provides huge advantage in terms of processing and hardware requirements. This also makes the system more fault tolerant. The robot team is very successful in achieving the goal even though several constraints were imposed on the system. These constraints included physical constraints, such as cable length, actuator constraints, communication limitations, and the limited degree of freedom of the robots.

The results obtained for cases where the targets are located close to the boundary of the workspace were also discussed. We will look into these cases in more detail. As future work, we also plan to train such systems using the GFS framework for scenarios with moving targets. We also plan to use this approach for other problems, such as team of UAVs collaboratively lifting an object to a target position.

## Data Availability Statement

The data related to this work are available on request to the corresponding author.

## Ethics Statement

Written informed consent was obtained from the individual(s) for the publication of any potentially identifiable images or data included in this article.

## Author Contributions

AS, KC, and OM: study conception and design, analysis and interpretation of results, and manuscript preparation. AS: data collection and training the model. All authors reviewed the results and approved the final version of the manuscript.

## Conflict of Interest

The authors declare that the research was conducted in the absence of any commercial or financial relationships that could be construed as a potential conflict of interest.
